# The Impact of a Human Papillomavirus Facebook-Based Intervention (#HPVVaxTalks) Among Young Black (African American and Sub-Saharan African Immigrants) Adults: Pilot Pre- and Poststudy

**DOI:** 10.2196/69609

**Published:** 2025-04-02

**Authors:** Adebola Adegboyega, Amanda Wiggins, Abubakari Wuni, Melinda Ickes

**Affiliations:** 1 College of Nursing, University of Kentucky, Lexington, KY, United States; 2 Department of Kinesiology and Health Promotion, College of Education, University of Kentucky, Lexington, KY, United States

**Keywords:** human papillomavirus vaccination, HPV vaccination, Facebook-based intervention, young Black adults, social media

## Abstract

**Background:**

Despite the availability of prophylactic human papillomavirus (HPV) vaccines, uptake remains suboptimal among young Black adults. Social media is a platform for the dissemination of health information and can be used to promote HPV vaccination among young Black adults.

**Objective:**

This study aimed to assess the impact of a Facebook-based intervention (#HPVVaxTalks), which consisted of 40 posts over 8 weeks in improving cognitive outcomes, reducing vaccine hesitancy, and increasing vaccine intention, and uptake among young Black adults aged 18-26 years.

**Methods:**

A pilot 1-group pre- and poststudy was conducted among 43 young Black adults who engaged in an 8-week Facebook intervention (#HPVVaxTalks). #HPVVaxTalks was developed in collaboration with a youth community advisory committee. Participants were actively recruited by research staff from community settings using flyers, and flyers were posted in public places in communities. Eligible participants were screened for eligibility and consented prior to study participation. Participants completed baseline surveys and were added to a Facebook page created for the study to receive intervention posts. Participants completed pre- and postdata on HPV knowledge, HPV vaccine knowledge, vaccine hesitancy, and vaccine uptake via REDCap (Research Electronic Data Capture) surveys distributed by email. Participants’ satisfaction with the intervention was collected via individual interviews. Data were analyzed using 2-tailed paired *t* tests and repeated measures analysis

**Results:**

Overall, 32 of the 43 (74%) participants completed the follow-up survey, and of the 23 participants who reported not having ever received the vaccine at baseline, 7 (30%) reported receiving the vaccine at follow-up. Participants demonstrated significant improvements in HPV knowledge and receiving the vaccine at follow-up. Participants demonstrated significant improvements in HPV knowledge (pre: mean 7.3, SD 4.2 and post: mean 11.1, SD 4.3; *P*=.004) and HPV vaccine knowledge (pre: mean 2.8, SD 2.5 and post: mean 4.7, SD 2.2; *P*=.003) and reduction in vaccine hesitancy (pre: mean 28.3, SD 4.2 and post: mean 29.9, SD 3.6; *P*=.007) after the intervention. However, there were no significant changes in other outcomes. Feedback from open-ended questions and qualitative interviews highlighted participants’ satisfaction with the intervention and its role in increasing HPV and HPV vaccine awareness.

**Conclusions:**

The findings from this study underscore the potential of social media platforms for health promotion among underrepresented populations and the importance of advocating for culturally appropriate interventions to improve HPV vaccination rates and reduce disparities.

## Introduction

### Background

Human papillomavirus (HPV) vaccination provides primary prevention against HPV-related diseases [1,2]. However, HPV vaccine uptake remains low for female participants and lower for male participants. Within 12 years of HPV vaccine introduction, infections with the 4 HPV types prevented by Gardasil decreased to 88% among 14‐ to 19-year-old female participants and to 81% among 20‐ to 24-year-old female participants in the United States [3]. According to the Centers for Disease Control and Prevention, only 21.5% of adults aged 18‐26 years received the recommended number of doses of the HPV vaccine [4]. Completion rates for the HPV vaccine are low in Black female participants (44.7% vs 57.9% White female participants) and lower among Black male participants (29.4% vs 26.6% among White male participants) aged 18‐26 years [5]. HPV vaccination is recommended for everyone through age 26 years if not adequately vaccinated when younger. Hence, Black young adults are a priority population to improve HPV vaccine uptake, given the low HPV vaccine uptake among this group [6,7].

In general, one of the most common barriers to HPV vaccination uptake is the lack of awareness of HPV and HPV infections. Research shows that sub-Saharan African immigrants have even less knowledge of HPV and HPV vaccination compared to White and Black American–born individuals [8-10]. The lack of knowledge results in misconceptions and the thinking that the HPV vaccine is unnecessary because individuals do not perceive themselves at risk of HPV infection [11]. In addition, the potential cost of the HPV vaccine series completion, perceptions of vaccine safety, and worry regarding anticipated side effects are barriers to the uptake of vaccine uptake for young adults [12]. In addition, system-level barriers such as limited access to vaccines, vaccine documentation requirements, and lack of strong provider recommendations may lead to missed vaccination opportunities [13-15]. Additionally, the African American population has been participants of historically unethical research, which may contribute to low vaccine uptake, medical mistrust, and misconceptions about vaccines [8,16,17].

Despite the high prevalence and complications that can occur from unresolved HPV infections, gaps in HPV-related cognition and HPV-related disparities exist for Black individuals. Catch-up HPV vaccination is recommended for young adults (up through age 26 years) who have not been previously vaccinated. However, few interventions have focused on Black young adults (18‐26 years), much of the research has explored parental decision-making and system-level interventions for HPV vaccination among adolescents [18-22]. Transition to young adulthood is a critical period to institute health-promoting behaviors [23]. As young adults transition to adulthood, they begin to explore their sexuality, including initiating sexual activity for the first time or engaging in riskier sexual behaviors than in the past. These behaviors increase the risk of acquiring sexually transmitted infections including HPV, providing a strong justification for health-promoting behavior such as catch-up HPV vaccination [24]. As Black young adults assume health care decision-making responsibility and independence, they may benefit from interventions to promote cognitive outcomes (HPV and HPV vaccine-related knowledge, benefits, severity, self-efficacy, risk perception), vaccine intention, and catch-up HPV vaccination.

Social media is increasingly becoming the preferred platform for the dissemination of health information and can be used to raise awareness about HPV and HPV vaccinations, therefore increasing HPV vaccination rates [25]. Social media platforms are multifaceted, and their features can be used to serve multiple purposes and reach a wide audience. Social media platforms include video and photo posting, blogs, polls, and other functions [26,27]. In the United States, Facebook is the single most popular social network with over 2 billion daily users, and 73% of Black adults have ever used Facebook [28,29]. In addition, 83% of adult social media users report visiting Facebook in a typical week, and on average, young adults aged 18 to 24 years spend about 11 minutes every day on Facebook [30].

Recent studies have used Facebook-based interventions to promote HPV vaccination among young adults [31-33]. For example, a technology-mediated intervention including private Facebook posts improved knowledge among undergraduate college students in the HPV vaccination intervention relative to those in the control condition [32]. Similarly, a sponsored advertisement campaign on Facebook targeted at young female participants aged 18‐26 years found that participants engaged with and were receptive to watching narrative-based health information videos on Facebook. Higher video engagement was associated with stronger intentions to talk with a health care professional, talk with friends or family, and vaccinate against HPV [31]. Despite the growing popularity of Facebook-based interventions, few have targeted young Black adults including sub-Saharan African immigrants. To fill this gap, we sought to leverage Facebook for an HPV vaccination intervention among young Black adults—a priority catch-up population with the highest burden of HPV. Thus, the objective of this study was to examine the impact of a Facebook-based intervention (#HPVVaxTalks) on HPV vaccination uptake and secondary outcomes among Black (African American and sub-Saharan African immigrants) young adults (18‐26 years) of an 8-week Facebook-based intervention. We hypothesize that young Black adults who complete the intervention will have improvements in HPV knowledge and HPV vaccine knowledge and a reduction in vaccine hesitancy after the intervention.

### Theoretical Framework

The theory of planned behavior states that the intention to perform a behavior is the strongest predictor of whether an individual engages in certain behaviors [34]. The theory of planned behavior has been used to understand and predict HPV vaccination rates by examining what constructs most correlate with people’s intentions to receive HPV vaccine. For example, in a sample of college students, more positive attitudes and increased subjective norms were strongly correlated with increased intention to receive complete doses of the HPV vaccine [35]. Knowledge and health beliefs are antecedents of intention and subsequent behavior. Hence, the theory of planned behavior provides a framework to understand individual actions by identifying beliefs relevant to individuals and groups and to develop interventions to change beliefs [34].

## Methods

### Study Designs and Participants

This was a 1-group pre- and post-Facebook–based intervention pilot study conducted in a southeastern state. We used a mixed methods approach by administering a pre- and postintervention survey to the participants as well as conducting follow-up interviews with a subset of participants.

Participants for the study were young Black (African American and sub-Saharan African immigrants) adults between 18 and 26 years, given the age for HPV vaccination catch-up through age 26 years. We used the STROBE (Strengthening the Reporting of Observation Studies in Epidemiology) checklist to guide the reporting of the study (Checklist 1).

### Intervention

**Figure 1.**
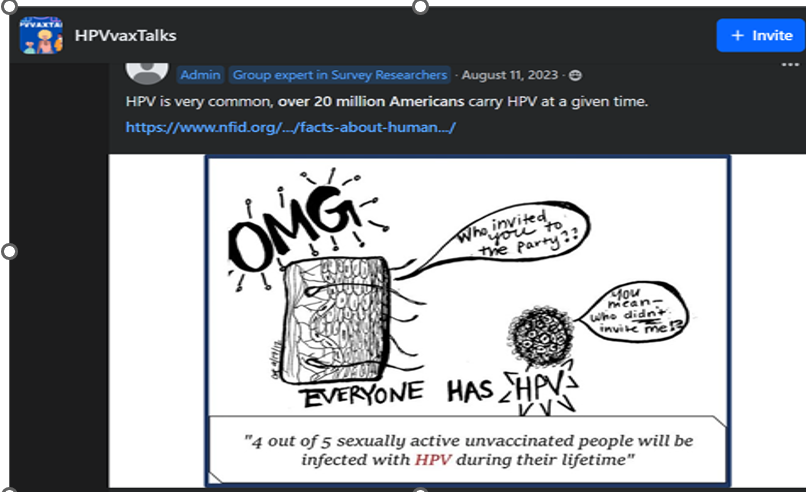
This is a sample of Facebook graphic posted as one of the messages for HPV vaccination promotion among young Black adults. Courtesy of National Foundation for Infectious Diseases [39].

A culturally appropriate Facebook-based intervention (#HPVVaxTalks) was developed in collaboration with a youth community advisory committee [36,37]. The research team created a library of evidenced-based Facebook posts, and a youth community advisory board reviewed the preliminary version of #HPVVaxTalks intervention materials and provided critiques and suggestions for refinement. The advisory board provided recommendations and input on different iterations of the posts for content preferences, priorities, user-friendliness, relevance, and cultural appropriateness [36]. To ensure posts were culturally appropriate, we followed strategies from Kreuter et al [38] including evidential, peripheral, linguistic, constituent-involving, and sociocultural strategies. Engagement with a youth community advisory board fostered the opportunity to discuss priorities reflecting the target group’s interest, ensure the appeal and relevance of the intervention, and draw on the experience of the target populations. In addition, we conducted a content validation of the intervention by obtaining experts’ feedback and participants’ satisfaction on the components of the #HPVVaxTalks for content usability, acceptability, relevance, and visual appeal to inform further refinements [37]. The intervention addressed general information on HPV, the importance of HPV vaccination, vaccine hesitancy, self-efficacy, HPV vaccination as a cancer prevention strategy, risk perception, perceived behavioral control, and subjective norm. Intervention contents were mapped to constructs of the theory of planned behavior, and important concepts informed by the literature and youth advisory board. The Facebook posts contained relevant images, graphics, memes, short texts, and information on where to receive the HPV vaccine in the community (see Figures1  2 for sample posts). We included embedded links to information from lead organizations (eg, American Cancer Society, American Sexual Health Association, and Centers for Disease Control and Prevention). The intervention consisted of 40 messages posted over 8 weeks, with 4 to 5 messages posted each week. One “secret” and “closed” Facebook group page solely for this study was created. Facebook was chosen as the platform for delivering HPV vaccination messages due to its features that allow for the creation of private groups. These groups enable only invited participants to see posts and engage in discussions, ensuring a level of privacy and confidentiality that can encourage open discussion [33]. Eligible participants were screened and consented, they received a link to complete the baseline survey and were invited to join #HPVVaxTalks Facebook group. Trained research staff posted intervention posts as scheduled (4 to 5 messages posted each week for 8 weeks) to promote engagement but not burden the participants with too much information. Enrolled participants were encouraged to turn on Facebook notification to know when posts are posted, view posts regularly, comment, like, and ask questions. The Facebook page was monitored throughout the intervention by the research team.

**Figure 2.**
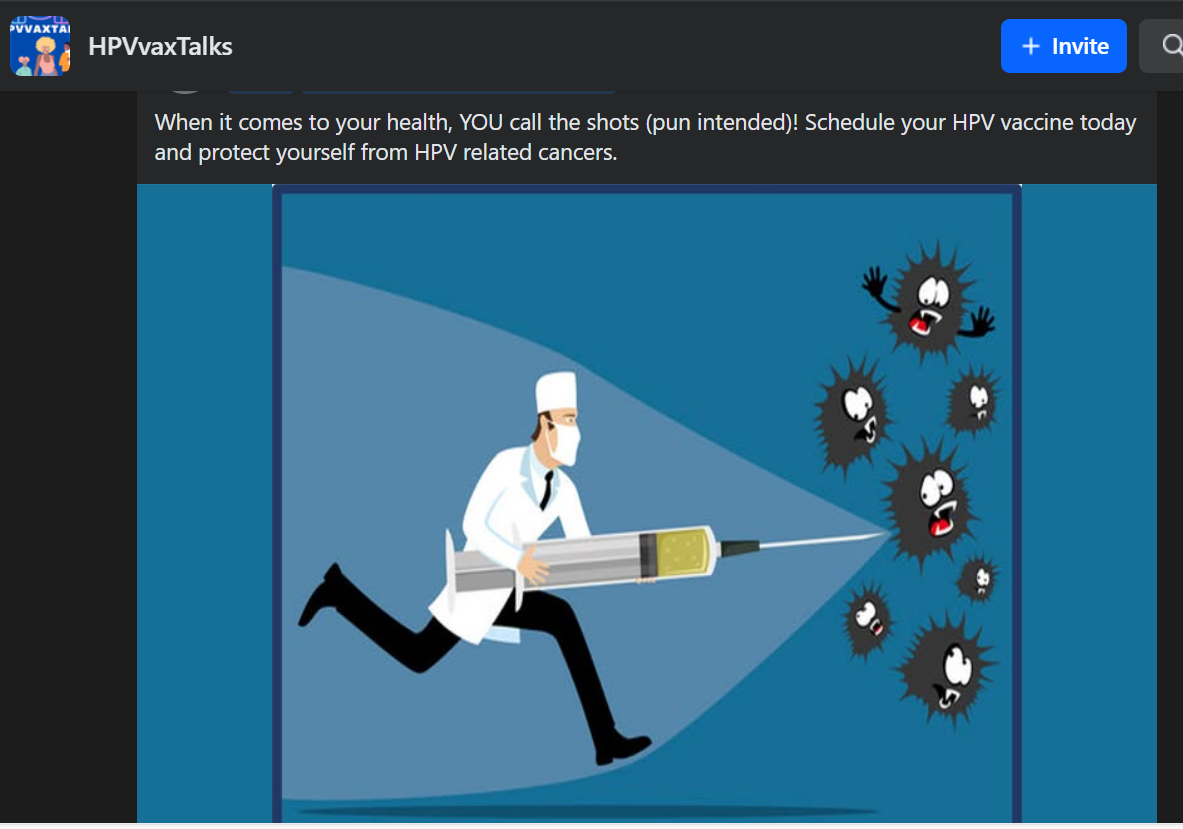
This is a sample of Facebook graphic posted as one of the messages for HPV vaccination promotion among young Black adults [39]. HPV: human papillomavirus.

### Recruitments

Black young adults aged 18‐26 years were recruited throughout a southeastern state between April 2023 and June 2024 using approved study flyers, snowballing, and word of mouth. In addition, research staff carried out active recruitment throughout community organizations that have a high client base of young adults. Study flyers with research team contact information were posted in public areas and community settings throughout the state. Flyers described the project and asked interested participants to contact the study team to learn more about the study. Interested participants were provided with additional information about the study and screened for eligibility. Study eligibility included the following: self-identification as African American and sub-Saharan African immigrants, age 18‐26 years, fluency in the English language, access to the internet, a Facebook account, and willingness to participate.

### Ethical Considerations

Prior to the start of the study, all study procedures and materials were reviewed and approved by the University of Kentucky Institutional Review Board (protocol 76589). Participation was voluntary, and all study participants provided written informed consent prior to participation in any study activities. Confidentiality of participant’s information was maintained at all times. Patient’s data were coded with a unique subject identifier, and all data deidentified prior to analysis. Participants were compensated with US $30 gift card from a retail store for completing the baseline survey and US $20 for postintervention surveys.

### Quantitative Data Collection

All research activities were completed remotely. Participants received REDCap (Research Electronic Data Capture; Vanderbilt University) links via emails to complete baseline and postintervention surveys. Quantitative data were collected using self-administered REDCap-based surveys at baseline and after the intervention (after 8 weeks). After the intervention, we repeated the items from the baseline survey and added open-ended questions to assess what participants liked about the intervention and ways for improvement. In addition, we assessed intervention satisfaction and asked if participants had received a dose of the HPV vaccination.

### Measures

#### Overview

Participants completed a self-administered survey including sociodemographic characteristics, HPV vaccination history, HPV knowledge items, HPV vaccine knowledge items, vaccine hesitancy measures, risk perception items, HPV severity, HPV vaccination benefits, and HPV vaccination self-efficacy. Valid and reliable measures were adapted from the literature [40-43].

#### Sociodemographic Characteristics

Sociodemographic information collected via self-report included age, sex, country of origin, education level, income, and health insurance coverage.

#### HPV Vaccination Uptake

HPV vaccination uptake was assessed among those who reported not being vaccinated at baseline. In the follow-up survey, participants were asked if they had received at least 1 dose of the HPV vaccine since enrolling in the study (yes or no).

#### HPV Knowledge

HPV knowledge items included 16 items assessing knowledge of HPV (including transmission, consequences, and risk factors). Response options were true or false or do not know, with “do not know” scored as incorrect [41,42]. A summative score was calculated with a potential range of 0‐16. The scale has high reliability (Cronbach α=0.84) among diverse populations [42], and Cronbach α for this study was 0.91.

#### HPV Vaccine Knowledge

HPV vaccine knowledge was assessed with an 8-item scale regarding the protection offered by HPV vaccines [41]. A summative score was calculated with a potential range of 0‐8. Cronbach α for this sample was 0.84.

#### Vaccine Hesitancy

Vaccine hesitancy was measured with a 9-item scale that captures general attitudes to vaccination [43], sample items include “the HPV vaccine is effective” and “the HPV vaccine is important for my health.” Responses options followed a 4-point Likert scale ranging from 1=strongly disagree to 4=strongly agree [43]. A summative score was calculated with a potential range of 9‐36, with lower scores indicating greater hesitancy. Cronbach α for this sample was 0.80.

#### HPV-Related Risk Perception

HPV-related risk perception was assessed with the item, “If I don’t get vaccinated for HPV, I think my chances of getting [HPV infection; HPV-related cancer; genital warts] sometime in the future would be,” with responses of 1=almost zero, 2=small, 3=moderate, 4=large, and 5=almost certain [40].

#### HPV Severity, Benefits, and Self-Efficacy

These constructs were assessed with items adapted from the literature [40]. HPV severity was assessed with 4 items (eg, being infected with HPV would have major consequences on my life), HPV benefits were assessed with 3 items (eg, getting vaccinated for HPV will help protect me from HPV infection), and self-efficacy was assessed with 3 items (eg, I feel confident in my ability to get vaccinated for HPV, even if it is expensive). Responses options for each item followed a 5-point Likert scale ranging from 1=strongly disagree to 5=strongly agree. Summative scores were calculated for each construct, with potential ranges of 4‐20, 3‐15, and 3‐15 for severity, benefits, and self-efficacy, respectively. Cronbach α for all scales exceeded 0.77.

#### HPV Vaccine Intention

Vaccine intention was assessed with an item, “I intend to get the HPV vaccine in the next 3 months?” Responses followed a 5-point Likert scale ranging from 1=strongly disagree to 5=strongly agree.

#### Intervention Satisfaction

Intervention satisfaction was assessed with items to measure usability (6 items; Cronbach α=0.93), acceptability (5 items; Cronbach α=0.92), and relevance of HPV posts or messages on Facebook (5 items; Cronbach α=0.92). Responses to each item followed a 5-point Likert scale ranging from 1=strongly disagree to 5=strongly agree, and summative scores were calculated for each construct separately [44,45].

### Data Analysis: Quantitative Data Analysis

Sociodemographic characteristics among those who completed the follow-up survey (completers) and those who did not (noncompleters) were examined using the 2-tailed *t* test, chi-square test of association, or Mann-Whitney *U* test, as appropriate.

Descriptive statistics were used to analyze vaccination uptake. Among those who had not received the HPV vaccine at baseline, unadjusted changes in outcomes (HPV-related knowledge, HPV vaccine knowledge, vaccine hesitancy, risk perception, severity, vaccination benefits, self-efficacy, and intention and vaccination uptake) were evaluated using the paired 2-tailed *t* test. These changes were also evaluated using repeated measures analysis, adjusting for age, sex, and ethnicity. The repeated measures analysis is exploratory due to the small size. Intervention satisfaction, specifically usability, acceptability, and relevance of the messages, was summarized descriptively using means and SDs. All analysis was conducted using SAS (version 9.4; SAS Institute Inc), with an α level of .05 throughout.

## Results

### Quantitative Findings: Sample Characteristics

**Figure 3.**
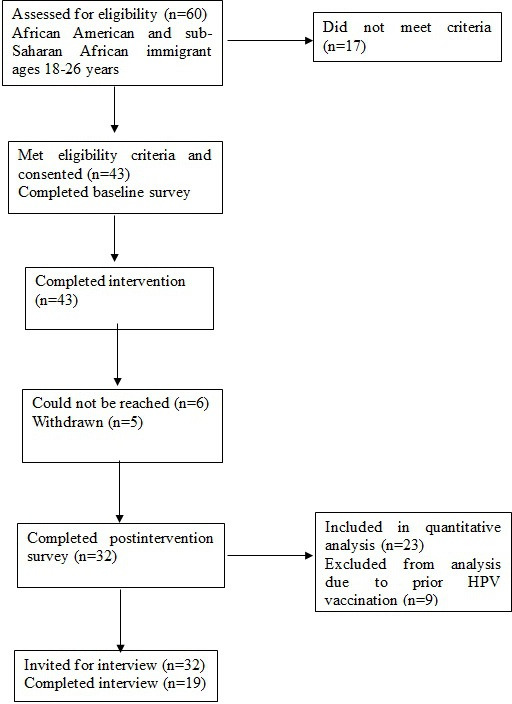
Flow participant diagram of the progress through the phases of a pre and post 1-group intervention (enrollment, baseline, completed intervention, follow-up, and data analysis). HPV: human papillomavirus.

In total, 43 young Black adults enrolled in the study, 32 of the 43 (74%) participants completed the follow-up survey, and 9 participants were ineligible for HPV vaccination because of prior HPV vaccination or other reasons not identified at the time of recruitment. Flow participant diagram through pre- and postintervention is shown in Figure 3. In the baseline sample of 43 participants, the average age was 21.7 (SD 2.7 years; Table 1). The majority were female (32/43, 74%), and more than half were African-born (25/43, 58%). Participants were well-educated, with nearly half having a college degree or beyond (20/33, 46%), and over three-quarters of participants had health insurance coverage (33/43, 77%). In comparing the completers and noncompleters of the follow-up survey, a higher proportion of completers were African-born (*P*=.002), had higher educational attainment (*P*=.02), and had higher income levels (*P*=.04). There was no difference in age, sex, health insurance coverage, or receipt of the HPV vaccine (at baseline) between completers and noncompleters.

**Table 1.** Descriptive summary of sociodemographic characteristics of young adults (18‐26 years).

	Full sample (N=43)	Completers (n=32)	Noncompleters (n=11)	*P* value
Age (years), mean (SD)	21.7 (2.7)	22.1 (2.9)	20.5 (1.6)	.09^a^
**Sex, n (%)**				.88^b^
Male	11 (26)	8 (25)	3 (27)	
Female	32 (74)	24 (75)	8 (73)	
**Country of origin, n (%)**				002^b^
US-born	18 (42)	9 (28)	9 (82)	
African-born	25 (58)	23 (72)	2 (18)	
**Education, n (%)**				.02^c^
High school or below	4 (9)	2 (6)	2 (18)	
Some college	19 (44)	12 (38)	7 (64)	
College graduate	13 (30)	11 (34)	2 (18)	
Master or higher	7 (16)	7 (22)	0 (0)	
**Household income (US $), n (%)**				.04^c^
Less than $25,000	14 (33)	11 (34)	3 (27)	
$25,000-$49,999	12 (28)	12 (38)	0 (0)	
$50,000-$79,999	7 (16)	5 (16)	2 (18)	
$80,000 or more	10 (23)	4 (13)	6 (55)	
**Health insurance coverage, n (%)**				.23^b^
Yes	33 (77)	26 (81)	7 (64)	
No	10 (23)	6 (18)	4 (36)	
**Ever recieved the human papillomavirus vaccine, n (%)**				.96^b^
Yes	12 (28)	9 (28)	3 (27)	
No or do not know	31 (72)	23 (72)	8 (73)	

a Two-tailed *t* test.

b Chi-square test of association.

c Mann-Whitney *U* test.

Among the 23 participants who completed the follow-up survey that reported not having ever received a dose of the HPV vaccine at baseline, 7 (30%) reported receipt of at least 1 dose of the HPV vaccine at follow-up. Among participants (n=23), there was a significant increase in HPV knowledge (*P*=.01) and HPV vaccine knowledge (*P*=.004; Table 2) in the unadjusted analysis. Based on a potential range of 0‐16, average HPV knowledge scores increased from 7.3 (SD 4.2) to 11.1 (SD 4.3) from baseline to follow-up. For HPV vaccine knowledge, based on a potential range of 0‐8, average scores increased from 2.8 (SD 2.5) on the baseline survey to 4.7 (SD 2.2) on the follow-up. Both of these changes remained statistically significant in the models adjusting for age, sex, and ethnicity (*P*=.004 and *P*=.003, respectively). There was also a significant change in HPV vaccine hesitancy. Scores on this scale increased from baseline (mean 28.3, SD 4.2) to follow-up (mean 29.9, SD 3.6; *P*=.02), which indicates a decrease in vaccine hesitancy. This change remained significant in the adjusted analysis (*P*=.007).

**Table 2.** Pre- and postchanges from baseline to follow-up among young adults (18‐26 years) who have not received the human papillomavirus (HPV) vaccine at baseline (n=23).

	Potential range	Baseline, mean (SD)	Postintervention, mean (SD)	Unadjusted *P* value	Adjusted *P* value^a^
HPV knowledge	0‐16	7.3 (4.2)	11.1 (4.3)	.01	.004
HPV vaccine knowledge	0‐8	2.8 (2.5)	4.7 (2.2)	.004	.003
HPV vaccine hesitancy	9‐36	28.3 (4.2)	29.9 (3.6)	.02	.007
HPV-related risk perception	1‐5	2.4 (1.1)	2.7 (1.1)	.44	.58
HPV severity	4‐20	16.5 (2.1)	16.5 (3.7)	.96	.93
HPV vaccination benefits	3‐15	12.3 (1.8)	13.1 (2.2)	.19	.12
HPV vaccination self-efficacy	3‐15	10.7 (2.1)	11.4 (2.6)	.34	.13
HPV vaccine intention	1‐5	2.7 (0.8)	2.6 (1.1)	.88	.90

a Models adjusted for age, ethnicity, and sex.

There were no significant changes in HPV severity, vaccination benefits, vaccination self-efficacy, or intention among those who had not received the HPV vaccine at baseline. Among the completers of the intervention, baseline vaccine hesitancy and risk perception scores were near the midpoint of the scales, while HPV severity, vaccination benefits, and vaccination self-efficacy were relatively high.

For intervention satisfaction, participants rated the usability high (eg, information was important, credible, helpful, etc) with an average score of 24.8 (SD 3.4) of a potential maximum of 30. Similarly, based on potential ranges of 0‐25, overall intervention acceptability (mean 20.0, SD 3.3; potential range 5‐25) and relevance of the posts (mean 20.1, SD 2.7) were high. The usability, acceptability, and relevance of the posts or messages were supported further by one-on-one interviews (n=19) and open-ended questions (n=32).

### Qualitative Data Collection

All participants who completed the postintervention assessments were invited for a follow-up interview via email. Follow-up one-on-one interviews were conducted with 19 participants to assess intervention satisfaction at 8 weeks (postintervention). The interviews were conducted on Zoom (Zoom Video Communications) by the first author (AA), a PhD-level qualitatively trained Black female researcher. Each interview began with an introduction, and the interview was guided by a semistructured interview guide developed by the research team. Each interview lasted 15 to 25 minutes. The interview questions were centered on how well the posts resonated with participants, how the intervention could be improved, and how the information learned from the intervention would be used. In addition, we asked if participants had received at least 1 dose of the HPV vaccination or scheduled an appointment. Participants were compensated with US $10 for interviews. Interviews were audio-recorded, transcribed verbatim, and deidentified for analysis.

### Qualitative Data Analysis

Data from the one-on-one interviews and open-ended questions were analyzed by 2 qualitative trained research team members (Adegboyega Adebola and Wuni Abubakari ). The researchers (AA and AW) read the transcripts and independently carried out a content analysis of the data. We did not use any qualitative software. Transcripts were coded line-by-line by the 2 researchers. Coding involved aggregating the data text into small categories of information and assigning a label to the code [46]. We used an iterative procedure to refine the codes and identify emergent themes until reaching saturation. Any discrepancies between results were discussed and resolved with the entire research team.

### Qualitative Findings

In total, 19 participants completed the interviews, 12 (63%) were female participants. Two main themes emerged from the data: (1) feedback and recommendation on the intervention and (2) perceived impact of the intervention on vaccination decision.

#### Theme 1: Feedback and Recommendation on the Intervention

Participants expressed strong appreciation for the visual content used in the intervention, particularly memes and graphics. Participants found these visuals to be effective in delivering clear and engaging messages about HPV and vaccination. One participant stated, “the use of memes and graphics made the information clear and easy to understand.” Another participant noted, *“*visuals really caught my attention and made me more interested in learning about HPV.” The participants positively noted the notification system for new posts and the use of incentives to encourage engagement. One participant noted that “I like the fact that I get notified of when new information is posted.” Opportunities for improvements suggested by participants included the promotion of more participant engagement, the use of additional platforms, and shorter intervention duration. One participant suggested, “You should make it into a group that everybody is active, maybe like text messaging, SMS, WhatsApp or something like a smaller group.” Another participant noted, “I think I will like it shorter for half of the time it was done, like four weeks.”

#### Theme 2: Perceived Impact of the Intervention on Vaccination Decision

Some participants who took the vaccine after the intervention reported that it significantly influenced their decision to vaccinate against HPV. Some participants indicated that the information provided through the intervention helped clarify misconceptions and increased their confidence in the vaccine’s necessity. One participant noted, “After learning more from the intervention, I decided to get vaccinated.” Another noted*,* “The intervention gave me the information I needed to make an informed decision about HPV vaccination.”

## Discussion

### Principal Findings

This study was a pilot 1-group pre- and post-Facebook–based intervention (#HPVVaxTalks) study among 43 young Black adults in a southeastern state. Participants in the 8-week intervention showed significant improvements in HPV knowledge and HPV vaccine knowledge and a reduction in vaccine hesitancy after the intervention. Participants were satisfied with the intervention, and it was well-received. Participants did not show significant changes in HPV severity, vaccination benefits, vaccination self-efficacy, or intention among those who had not received the HPV vaccine at baseline.

The intervention led to HPV vaccination uptake, with 7 of 23 (30%) reporting that they have received at least 1 dose of the HPV vaccine at 8 weeks follow-up. This is a noteworthy result, given that the intervention targeted a high-risk group. This finding is in line with a study where 30% of Korean-American female participants aged 21 to 29 years who participated in a mobile health intervention received the first dose of the HPV vaccine after the intervention of a text messaging intervention to promote HPV vaccination [47]. Our finding highlights the impact of culturally appropriate intervention in promoting HPV vaccination among previously unvaccinated individuals. However, the low vaccination rates and no significant changes in HPV vaccine intention reported from our study show that this intervention had limited influence on HPV vaccination outcomes. According to the theory of planned behavior, the intention to perform a behavior is the strongest predictor of whether an individual engages in certain behaviors. The intention to engage in vaccination is determined by a person’s beliefs, attitudes, and subjective norms within a specific context [34]. Hence, future interventions should use multicomponent interventions to address young adults’ HPV vaccine–related beliefs, attitudes, and subjective norms. Multicomponent interventions should include effective patient-provider communication and HPV vaccine recommendations as a cancer prevention tool. In addition, there is a need for comprehensive social media campaigns to address myths and misinformation related to HPV targeted at young adults, given their wide reach and influence among young adults.

This study found a significant increase in HPV knowledge and HPV vaccine knowledge in the pre- and postintervention. The findings from this study are congruent with a social media intervention study where there was a statistically significant increase in knowledge after the intervention [48]. Furthermore, qualitative postintervention interviews with study participants supported the findings that our social media intervention increased their awareness and knowledge of HPV, particularly regarding transmission risks and preventive measures. This is in line with a similar study on mobile health interventions among unvaccinated youths, which showed improvement in HPV-related knowledge, vaccination intention, or vaccine initiation in a postintervention [49]. However, the findings from our study are inconsistent with a Twitter campaign that reported no significant changes in HPV knowledge after the campaign [50]. However, knowledge alone is insufficient for increasing HPV vaccination rates, as other factors beyond knowledge deficiency influence HPV vaccination decisions.

Our study found a significant reduction in HPV vaccine hesitancy. This finding supports the use of a Facebook-based intervention to decrease HPV vaccine hesitancy among young Black adults. A large-scale study across 67 countries showed that vaccine hesitancy can partly be attributed to a lack of confidence in vaccine safety, perceptions that vaccines do not work, distrust of information, perceived low risks of vaccine-preventable diseases, as well as a lack of trust in health care providers, authorities, and pharmaceutical companies [51]. Research has shown that interventions that support risk communication, community engagement, use of trained vaccine champions, tailored communication campaigns, and increased confidence in vaccines tend to reduce HPV vaccine hesitancy [52]. For this pilot study, we used a community-engaged approach by engaging with a youth advisory board for the development of #HPVVaxTalks intervention, which improved its cultural appropriateness for young Black adults. Future studies could use young adults as vaccine champions to deliver training and advocacy tailored to their communities to positively impact social norms [52].

Our study found no significant differences in HPV self-efficacy, intention to vaccinate, perceived severity, and perceived vaccination benefits. This may be because of the small sample size and relatively high baseline values for these variables. Future research with large sample size and longitudinal approach is warranted to test whether a Facebook-based intervention is effective in promoting significant change in HPV self-efficacy, intention to vaccinate, perceived severity, perceived vaccination benefits, and HPV vaccination uptake.

The intervention piloted in this study was well-received, with participants rating its usability, acceptability, and relevance highly. This study reiterates the importance of including the target population in formative research to promote usability, acceptability, and relevance to the target population. These findings are supported by research that reports that young adults are open to using social media to seek out health information and learn more about preventative health behaviors [32,48,50]. Using social media platforms could be an avenue and platform for delivering crucial information to reach young Black adults on a larger scale.

Participants reported that the intervention positively influenced their perception of the HPV vaccine’s necessity and encouraged them to consider vaccination. These findings are supported by research that reports that young Black adults can benefit from social media interventions and seek out correct health information from social media platforms. Leader et al [31] showed that young adults were receptive to watching narrative-based health information videos on social media. Based on suggested opportunities for improvement, future research may explore shorter intervention duration to prevent attrition and use other social media avenues that may be relevant for health promotion and engagement for African American and sub-Saharan African immigrant Black young adults.

### Implications for Practice or Research

The intervention’s effect on HPV-related knowledge, vaccine knowledge, and vaccine hesitancy demonstrates its efficacy. Researchers can extend these findings by undertaking larger-scale research with varied groups and using other social media platforms to demonstrate the intervention’s reproducibility and generalizability. Follow-up studies could determine whether knowledge increases, and behavioral improvements are sustainable over time. To determine if intervention effects persist beyond study completion and if participants complete the HPV vaccination series, longitudinal research is needed to monitor participants’ HPV vaccination series completion. In addition, further research can determine which parts of the intervention (eg, specific message kinds and frequency of exposure) are most important to its effectiveness.

Tailored and community-driven social media interventions can be implemented to increase overall community uptake and support of the intervention and may improve health outcomes among minoritized and underserved populations. Health care professionals can use social media platforms to share evidence-based information regarding HPV and HPV vaccination. The intervention’s success in increasing knowledge and vaccination shows that such initiatives could be beneficial on a larger scale. Messages can be tailored to young Black adults who are active on social media while taking into consideration cultural contexts and incorporating strategies to increase engagement and the effectiveness of future interventions. The usability, acceptability, and relevance of the intervention point to its usefulness in informing future large-scale Facebook-based studies.

### Strengths and Limitations

Strengths of this study include the use of theory-driven intervention developed by a youth community advisory board, the use of a social media intervention, and a mixed methods approach. There are limitations to this study to consider. The sample is small and limited to those who had access to Facebook, and results are not generalizable to all young Black adults. The use of convenience sampling could have led to a homogenous participant pool and limited the diversity of perspectives. We collected self-reported measures, which could have social desirability bias. We also experienced a high attrition rate in the study. Future studies should oversample and send multiple routine reminders about follow-ups to minimize attrition. Another limitation of this study is the lack of a comparable control group. Similar to many pilot studies, the study was not powered, but provides a foundation for future studies, to be able to pilot-test the intervention and posts.

### Conclusions

This study highlights significant improvements in HPV knowledge and HPV vaccine knowledge and a reduction in HPV vaccine hesitancy following a Facebook-based intervention among young Black adults. The findings from this study contribute to available research on the use of Facebook intervention among young Black adults. Intervention addressing knowledge gaps and improving HPV hesitancy among potential vaccine recipients is pivotal to promoting vaccine uptake. More research is needed to assess the type of messages and intervention dose that can most effectively facilitate changes in HPV perceived severity, vaccination benefits, self-efficacy, and intention to vaccinate to promote HPV vaccination behavior among young Black adults.

## Supplementary material

10.2196/69609 Checklist 1 STROBE (STrengthening the Reporting of OBservational studies in Epidemiology) statement—checklist of items that should be included in reports of cohort studies.

## Abbreviations

HPV: human papillomavirus
REDCap: Research Electronic Data Capture
STROBE: Strengthening the Reporting of Observation Studies in Epidemiology
